# Supplementary material for: Mapping causal links between prefrontal cortical regions and intra-individual behavioral variability
Source: Nat Commun. 2024 Jan 2;15:140. doi: 10.1038/s41467-023-44341-5 (PMC10762061; doi:10.1038/s41467-023-44341-5)
Supplement: Supplementary file 1 — Supplementary Material [file 41467_2023_44341_MOESM1_ESM.pdf]

Supplementary Material

Computational background of Fig. 7

We assume that the evidence is constantly accumulated with time but the rate of evidence accumulation varies among trials even within cC trials. We denote the highest and lowest rates of evidence accumulation in cC trials in control monkeys by *a* and *b*, respectively (*a* > *b*). We assume that the decision threshold remains constant and does not vary among trials. We denote the decision threshold in control monkeys by D_{con}. Then,

the shortest RT is $\frac{D_{con}}{a}$

the longest RT is $\frac{D_{con}}{b}$

We consider the difference between the longest and shortest RT, which we call RT difference (denoted as RTD_{con}). It is drawn from the above two formula:

the RT difference (RTD_{con}) is $\frac{D_{con}\times(a-b)}{a\times b}$ (Equation 1)

We then consider how the RT difference changes when the rate of evidence accumulation and the decision threshold change by a lesion. We consider two cases; one in which the rate of evidence accumulation decreases divisionally by the lesion and the other in which the rate of evidence accumulation decreases subtractively by the lesion. We denote the decision threshold in the lesined monkeys by D_{les}.

1) Divisive case

We assume that the rate of evidence accumulation decreases by a lesion divisionally (divided by $c, c > 1$). Then, in the lesioned monkeys,

the shortest RT is $\frac{D_{les}\times c}{a}$

the longest RT is $\frac{D_{les}\times c}{b}$

the RT difference (RTD_{les}) is $\frac{D_{les}\times c\times(a-b)}{a\times b}$ (Equation 2a)

By combining the equations 1 and 2a,

$RTD_{les} - RTD_{con}$

$$= \frac{D_{les} \times c \times (a-b)}{a \times b} - \frac{D_{con} \times (a-b)}{a \ \times b}$$

$$= (\frac{D_{les}}{D_{con}} - \frac{1}{c}) \times \frac{D_{con} \times c \times (a-b)}{a \times b}$$

Then,

$RTD_{les} < RTD_{con}$ if $\frac{D_{les}}{D_{con}} < \frac{1}{c}$ (as in Figs. 7a and 7b)

$RTD_{les} > RTD_{con}$ if $\frac{D_{les}}{D_{con}} > \frac{1}{c}$ (as in Figs. 7c and 7d)

2) Subractive case

We assume that the rate of evidence accumulation decreases by a lesion subtractively (subtracted by $c, c > 0$). Then, in the lesioned monkeys,

the shortest RT is $\frac{D_{les}}{(a-c)}$

the longest RT is $\frac{D_{les}}{(b-c)}$

the RT difference is $\frac{D_{les} \times (a-b)}{(a-c) \times (b-c)}$ (Equation 2b)

By combining the equations 1 and 2b,

$$RTD_{les} - RTD_{con}$$

$$= \frac{D_{les} \times (a-b)}{(a-c) \times (b-c)} - \frac{D_{con} \times (a-b)}{a \times b}$$

$$= (\frac{D_{les}}{D_{con}} - \frac{(a-c) \times (b-c)}{a \times b}) \times \frac{D_{con} \times c \times (a-b)}{a \times b}$$

Then,

$RTD_{les} < RTD_{con}$ if $\frac{D_{les}}{D_{con}} < \frac{(a-c) \times (b-c)}{a \times b}$

$RTD_{les} > RTD_{con}$ if $\frac{D_{les}}{D_{con}} > \frac{(a-c) \times (b-c)}{a \times b}$

Thus, in either the divisive or subtractive case, if the reduction in decision threshold by the lesion is larger as compared with that in the rate of evidence accumulation (as in Figs. 7a and 7b), the RT difference in the lesioned monkeys decreases. The results in the ACC-lesioned and DLPFC-lesioned groups can be explained by this scheme. On the contrary, if the lesion causes no or little reduction in decision threshold, the RT difference increases, especially when the rate of evidence accumulation is significantly reduced (as in Figs. 7c and 7d). The results in the OFC-lesioned and PCC-lesioned groups can be explained by this latter schema.

Computational background of Fig. S3

cE vs. cC: The rate of evidence accumulation is expected to be larger in correct trials than that in error trials. We denote the mean rate of evidence accumulation in correct and error trials by a and b, respectively ($a > b$). We compare the RT difference in error trials with the RT difference in correct trials in two cases: one in which the variance in the rate of evidence accumulation is proportional to the mean rate of evidence accumulation (proportional case) and the other in which the variance in rate of evidence accumulation is constant regardless of the mean rate (constant case).

1) Proportional case

We denote the difference between the largest and smallest rates by 2v in units of the mean rate. Then, the largest and smallest rates in correct trials are $(1 + v) \times a$ and $(1 - v) \times a$, respectively, and the largest and smallest rates in error trials are $(1 + v) \times b$ and $(1 - v) \times b$, respectively ($0 < v < 1$). We denote the RT difference in correct trials in control monkeys by RTD_{con-c} and that in error trials in control monkeys by RTD_{con-e}.

In correct trials in control monkeys,

the shortest RT is $\frac{D_{con}}{a\times (1+v)}$

the longest RT is $\frac{D_{con}}{a\times(1-v)}$

RTD_{con-c} is $\frac{2v\times D_{con}}{a\times(1+v)\times(1-v)}$ (Equation 3)

In error trials in control monkeys,

the shortest RT is $\frac{D_{con}}{b\times(1+v)}$

the longest RT is $\frac{D_{con}}{b\times(1-v)}$

RTD_{con-e} is $\frac{2v\times D_{con}}{b\times(1+v)\times(1-v)}$ (Equation 4)

By combining the equations 3 and 4,

$$RTD_{con-e} - RTD_{con-c}$$

$$= \frac{2v \times D_{con}}{b \times (1+v) \times (1-v)} - \frac{2v \times D_{con}}{a \times (1+v) \times (1-v)}$$

$$= \frac{(a-b) \times 2v \times D_{con}}{a \times b \times (1+v) \times (1-v)}$$

> 0 (as shown in Figs. S3a and S3b)

2) Constant case

We denote the difference between the largest and smallest rates by 2v. Then, the largest and smallest rates in correct trials are $a + v$ and $a - v$, respectively, and the largest and smallest rates in error trials are $(b + v)$ and $(b - v)$, respectively ($0 < v < b$).

In correct trials in control monkeys,

the shortest RT is $\frac{D_{con}}{a+v}$

the longest RT is $\frac{D_{con}}{a-v}$

RTD_{con-c} is $\frac{2v \times D_{con}}{(a+v)\times(a-v)}$ (Equation 5)

In error trials in control monkeys,

the shortest RT is $\frac{D_{con}}{b+v}$

the longest RT is $\frac{D_{con}}{b-v}$

RTD_{con-e} is $\frac{2v\times D_{con}}{(b+v)\times(b-v)}$ (Equation 6)

By combining the equations 5 and 6,

$RTD_{con-e} - RTD_{con-c}$

$$= \frac{2v \times D_{con}}{(b+v) \times (b-v)} - \frac{2v \times D_{con}}{(a+v) \times (a-v)}$$

$$= \frac{(a+v) \times (a-v) - (b+v) \times (b-v) \times D_{con}}{(a+v) \times (a-v) \times (b+v) \times (b-v)}$$

> 0

Thus, in either the proportional or constant case, the RT difference in error trials is larger than the RT difference in correct trials.

ACC-lesioned group vs. Control group: We then consider how the difference between the RT difference in cE trials and the RT difference in cC trials changes when the decision threshold changes by ACC lesion. We here focus on ACC lesion because a consistent conclusion about the lesion's effect on the difference between the RT difference in cE trials and the RT difference in cC trials across indexes of varaibility (RT-COV and SD) was obtained only for ACC lesion. We denote the RT difference in correct trials in ACC-lesioned monkeys by RTD_{acc-c} and that in error trials in ACC-lesioned monkeys by RTD_{acc-e}, respectively. As the model consideration of the effects of ACC lesion on the RT difference in correct trials indicated that the change in the rate of evidence accumulation by ACC lesion is negligible as compared with the change in the decision threshold by ACC lesion, we here neglect the change in the rate of evidence accumulation by ACC lesion for the sake of simplicity.

As we assume that the evidence is accumulated linearly along time, the RT difference, either that in correct trials or that in error trials, will decrease after ACC lesion in proportion to the reduction in decision threshold.

$$RTD_{acc-c} = \frac{D_{acc}}{D_{con}} \times RTD_{con-c} \quad \text{(Equation 7)}$$

$$RTD_{acc-e} = \frac{D_{acc}}{D_{con}} \times RTD_{con-e} \quad \text{(Equation 8)}$$

By combining the equations 7 and 8,

$$RTD_{acc-e} - RTD_{acc-c} = \frac{D_{acc}}{D_{con}} \times (RTD_{con-e} - RTD_{con-c})$$

Thus, the difference between the RT difference in cE trials and the RT difference in cC trials will also decrease after ACC lesion in proportion to the ratio of the decision threshold in ACC-lesioned monkeys to that in control monkeys.

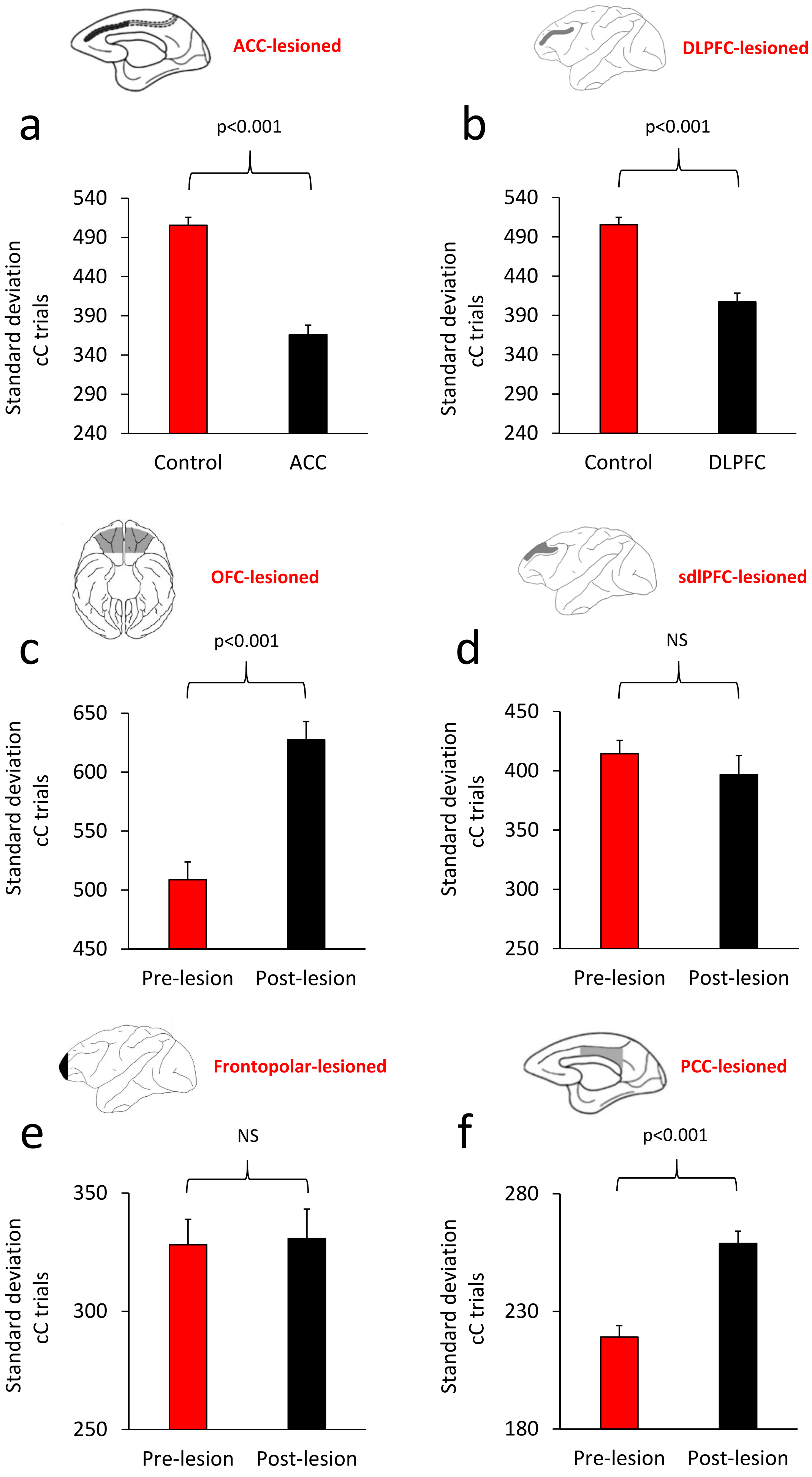

ACC-lesioned
DLPFC-lesioned
a
p<0.001
540
490
440
390
340
290
240
Standard deviation
cC trials
Control
ACC
b
p<0.001
540
490
440
390
340
290
240
Standard deviation
cC trials
Control
DLPFC
OFC-lesioned
sdlPFC-lesioned
c
p<0.001
650
600
550
500
450
Standard deviation
cC trials
Pre-lesion
Post-lesion
d
NS
450
400
350
300
250
Standard deviation
cC trials
Pre-lesion
Post-lesion
Frontopolar-lesioned
PCC-lesioned
e
NS
350
300
250
Standard deviation
cC trials
Pre-lesion
Post-lesion
f
p<0.001
280
230
180
Standard deviation
cC trials
Pre-lesion
Post-lesion

Figure S1. Standard deviation of response time in correct trials.

The standard deviation of response time (RT-SD) is shown for correct (cC) trials, in the same format as those in Figs. 2 and 3. (**a**) Anterior cingulate cortex (ACC). (**b**) Dorsolateral prefrontal cortex (DLPFC). (**c**) Orbitofrontal cortex (OFC). (**d**) Superior dorsal-lateral prefrontal cortex (sdlPFC). (**e**) Frontopolar cortex. (**f**) Posterior cingulate cortex (PCC).

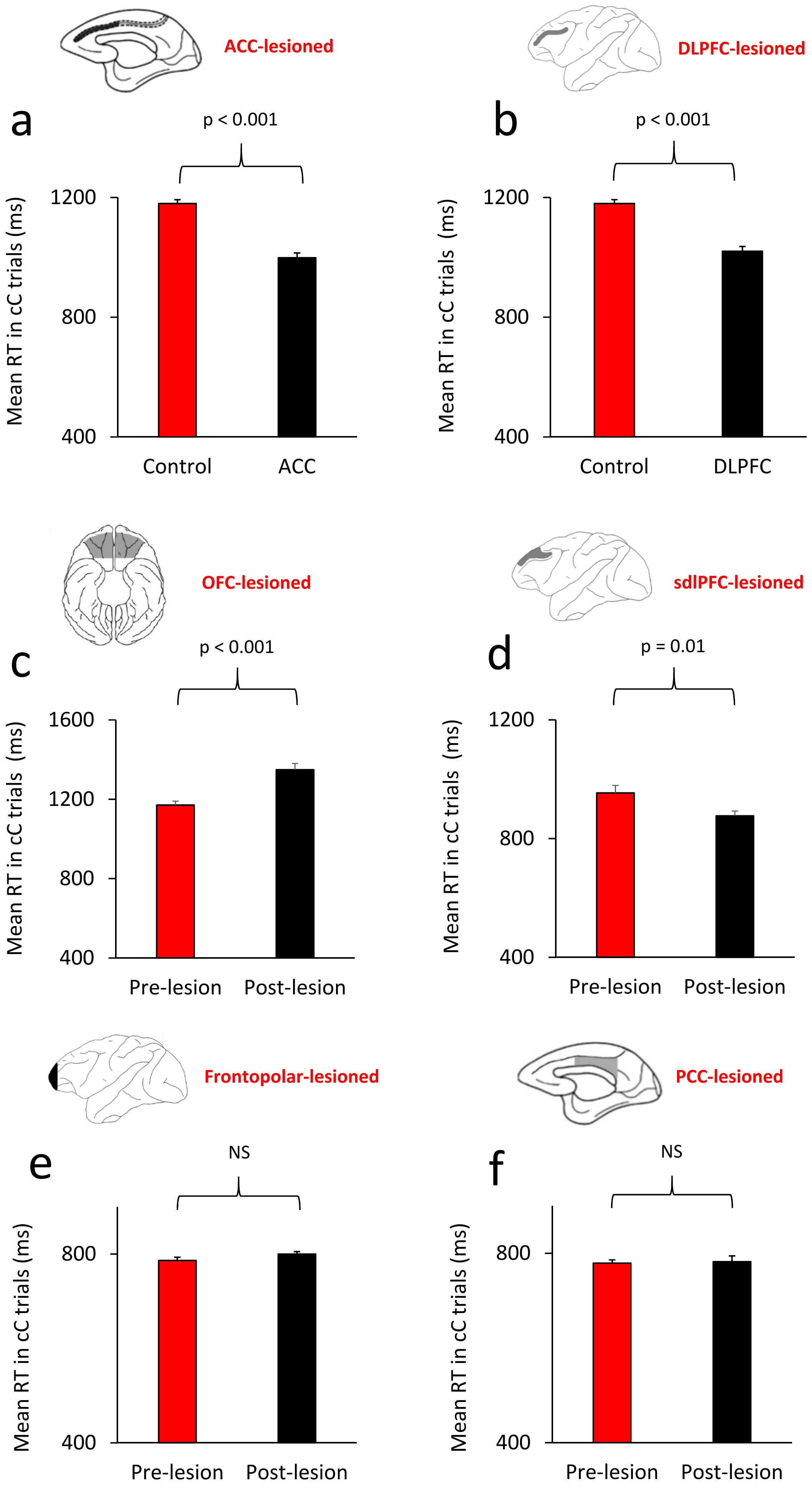

ACC-lesioned
a
p < 0.001
Mean RT in cC trials (ms)
1200
800
400
Control
ACC
DLPFC-lesioned
b
p < 0.001
Mean RT in cC trials (ms)
1200
800
400
Control
DLPFC
OFC-lesioned
c
p < 0.001
Mean RT in cC trials (ms)
1600
1200
800
400
Pre-lesion
Post-lesion
sdlPFC-lesioned
d
p = 0.01
Mean RT in cC trials (ms)
1200
800
400
Pre-lesion
Post-lesion
Frontopolar-lesioned
e
NS
Mean RT in cC trials (ms)
800
400
Pre-lesion
Post-lesion
PCC-lesioned
f
NS
Mean RT in cC trials (ms)
800
400
Pre-lesion
Post-lesion

Figure S2. Mean response time in correct trials.

The mean response time (RT) is shown for correct (cC) trials, in the same format as those in Figs 2 and 3. (**a**) Anterior cingulate cortex (ACC). (**b**) Dorsolateral prefrontal cortex (DLPFC). (**c**) Orbitofrontal cortex (OFC). (**d**) Superior dorsal-lateral prefrontal cortex (sdlPFC). (**e**) Frontopolar cortex. (**f**) Posterior cingulate cortex (PCC).

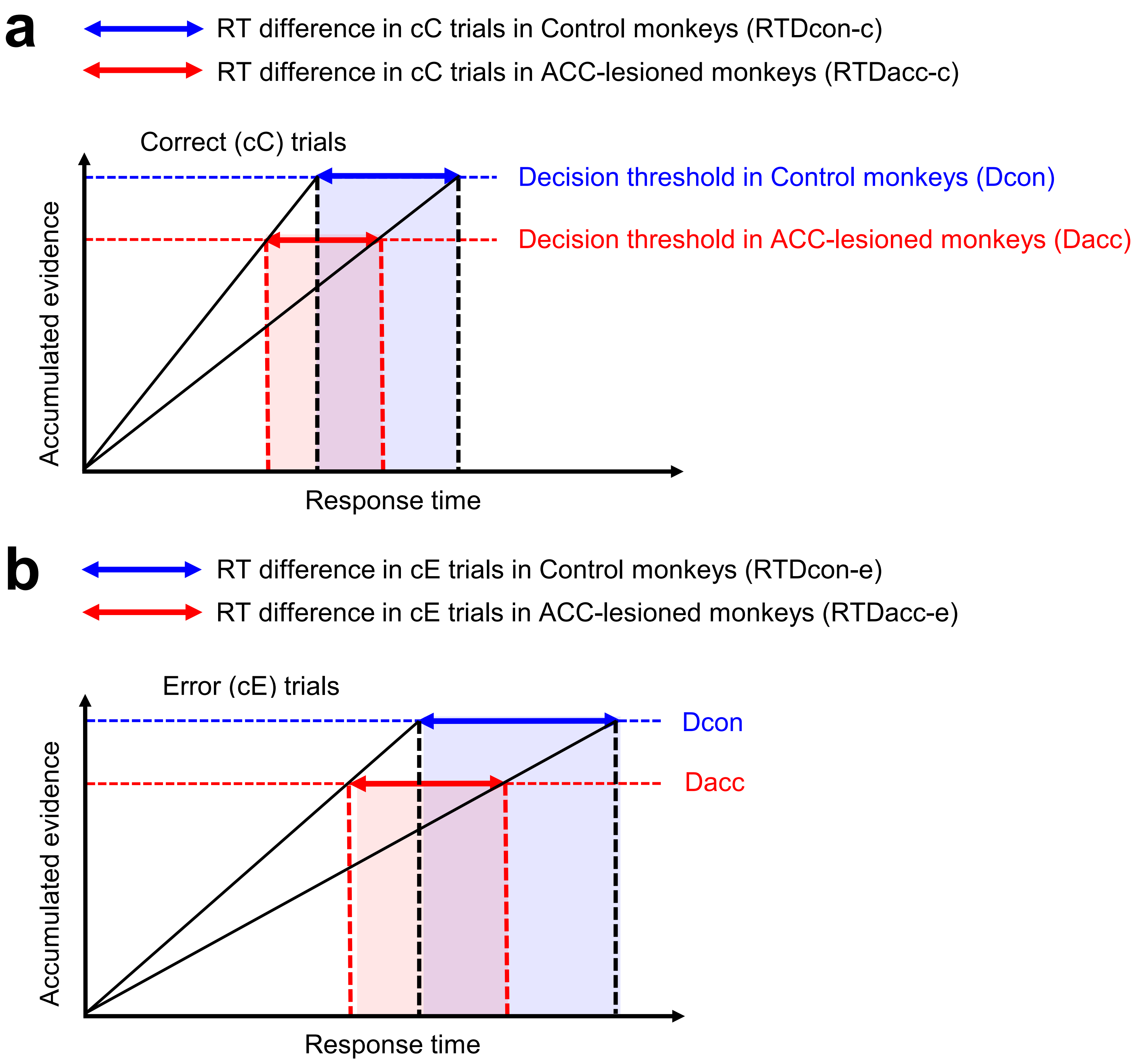
a
RT difference in cC trials in Control monkeys (RTDcon-c)
RT difference in cC trials in ACC-lesioned monkeys (RTDacc-c)
Correct (cC) trials
Decision threshold in Control monkeys (Dcon)
Decision threshold in ACC-lesioned monkeys (Dacc)
Accumulated evidence
Response time
b
RT difference in cE trials in Control monkeys (RTDcon-e)
RT difference in cE trials in ACC-lesioned monkeys (RTDacc-e)
Error (cE) trials
Dcon
Dacc
Accumulated evidence
Response time

Figure S3. The model depicting the consequence of decision threshold change on RT variability in correct and error trials.

The performance of the drifting model in cC trials (**a**) and cE trials (**b**). Two evidence accumulation lines are shown in each graph, for the cases with the highest and lowest rates of evidence accumulation. The difference in RT between the two evidence accumulation lines (the distance between the same-color vertical dashed lines) is shown as the red and blue bidirectional horizontal arrows (and corresponding shaded areas) for the ACC-lesioned and control monkeys, respectively. We denote the RT difference in correct and error trials in the Control group by RTD_{con-c} and RTD_{con-e}, respectively, and the RT difference in correct and error trials in the ACC-lesioned group by RTD_{acc-c} and RTD_{acc-e}, respectively. The model shows that the RT difference in error trials is larger than that in correct trials for both the Control and ACC-lesioned monkeys (RTD_{con-e}>RTD_{con-c}, and RTD_{acc-e}>RTD_{acc-c}). The model also shows that the RT difference in the ACC-lesioned monkeys is smaller than that in the Control monkeys for both correct and error trials ($RTD_{acc-c} < RTD_{con-c}$, and RTD_{acc-e}<RTD_{con-e}), and consequently the magnitude of the RT difference between error and correct trials is also smaller in the ACC-lesioned monkeys than in the Control monkeys ($RTD_{acc-e} - RTD_{acc-c} < RTD_{con-e} - RTD_{con-c}$). Refer to Supplemental material: 'Computational background of Fig. S3' for the computational background.

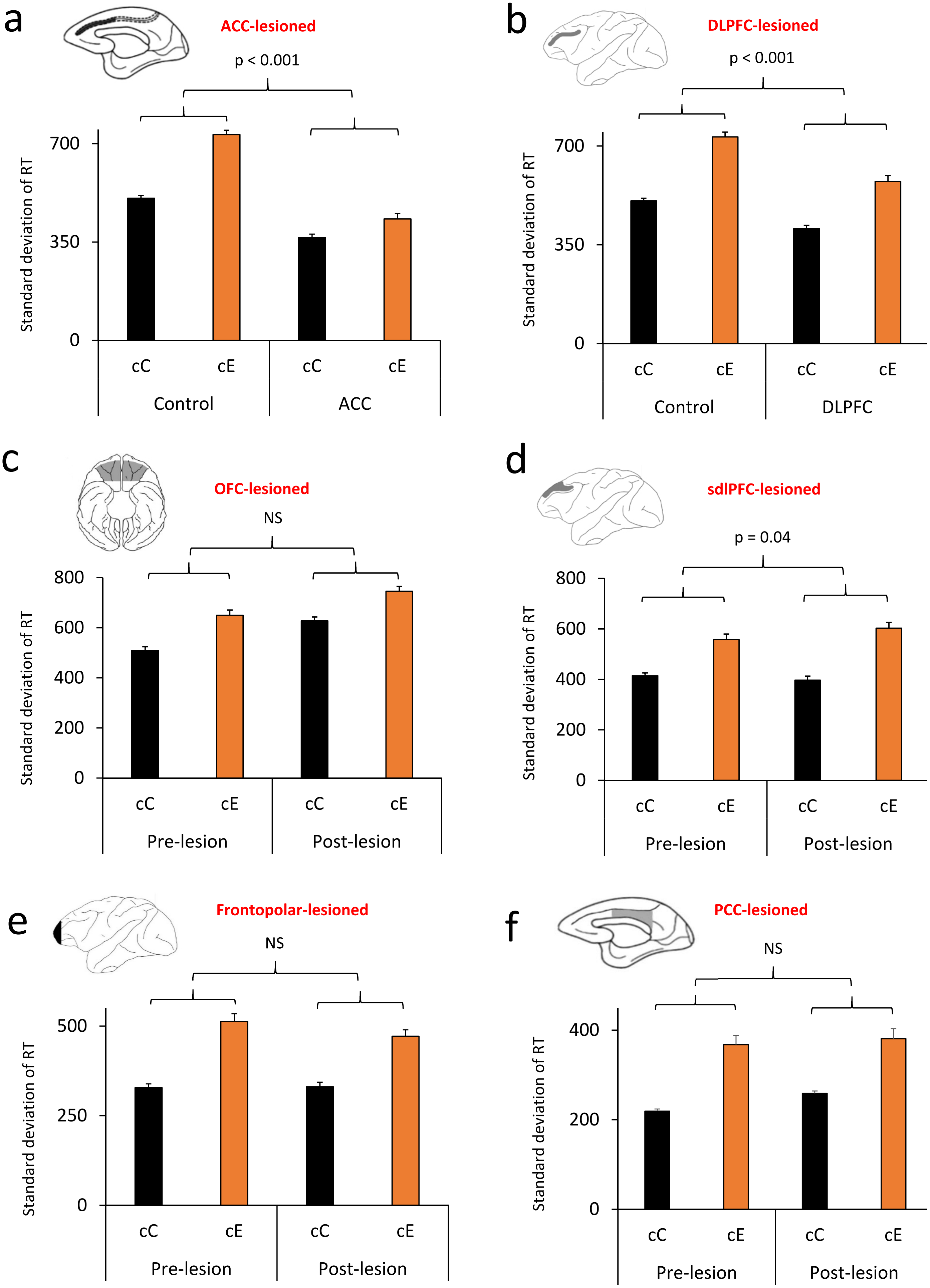

a
ACC-lesioned
p < 0.001
Standard deviation of RT
700
350
0
cC
cE
cC
cE
Control
ACC
b
DLPFC-lesioned
p < 0.001
Standard deviation of RT
700
350
0
cC
cE
cC
cE
Control
DLPFC
c
OFC-lesioned
NS
Standard deviation of RT
800
600
400
200
0
cC
cE
cC
cE
Pre-lesion
Post-lesion
d
sdlPFC-lesioned
p = 0.04
Standard deviation of RT
800
600
400
200
0
cC
cE
cC
cE
Pre-lesion
Post-lesion
e
Frontopolar-lesioned
NS
Standard deviation of RT
500
250
0
cC
cE
cC
cE
Pre-lesion
Post-lesion
f
PCC-lesioned
NS
Standard deviation of RT
400
200
0
cC
cE
cC
cE
Pre-lesion
Post-lesion

Figure S4. Consequence of selective brain lesions on standard deviation of RT in error trials.

The standard deviation of response time (RT-SD) is shown in correct (cC) and error (cE) trials, in the same format as those in Fig. 4. (**a**) Anterior cingulate cortex (ACC). (**b**) Dorsolateral prefrontal cortex (DLPFC). (**c**) Orbitofrontal cortex (OFC). (**d**) Superior dorsal-lateral prefrontal cortex (sdlPFC). (**e**) Frontopolar cortex. (**f**) Posterior cingulate cortex (PCC).

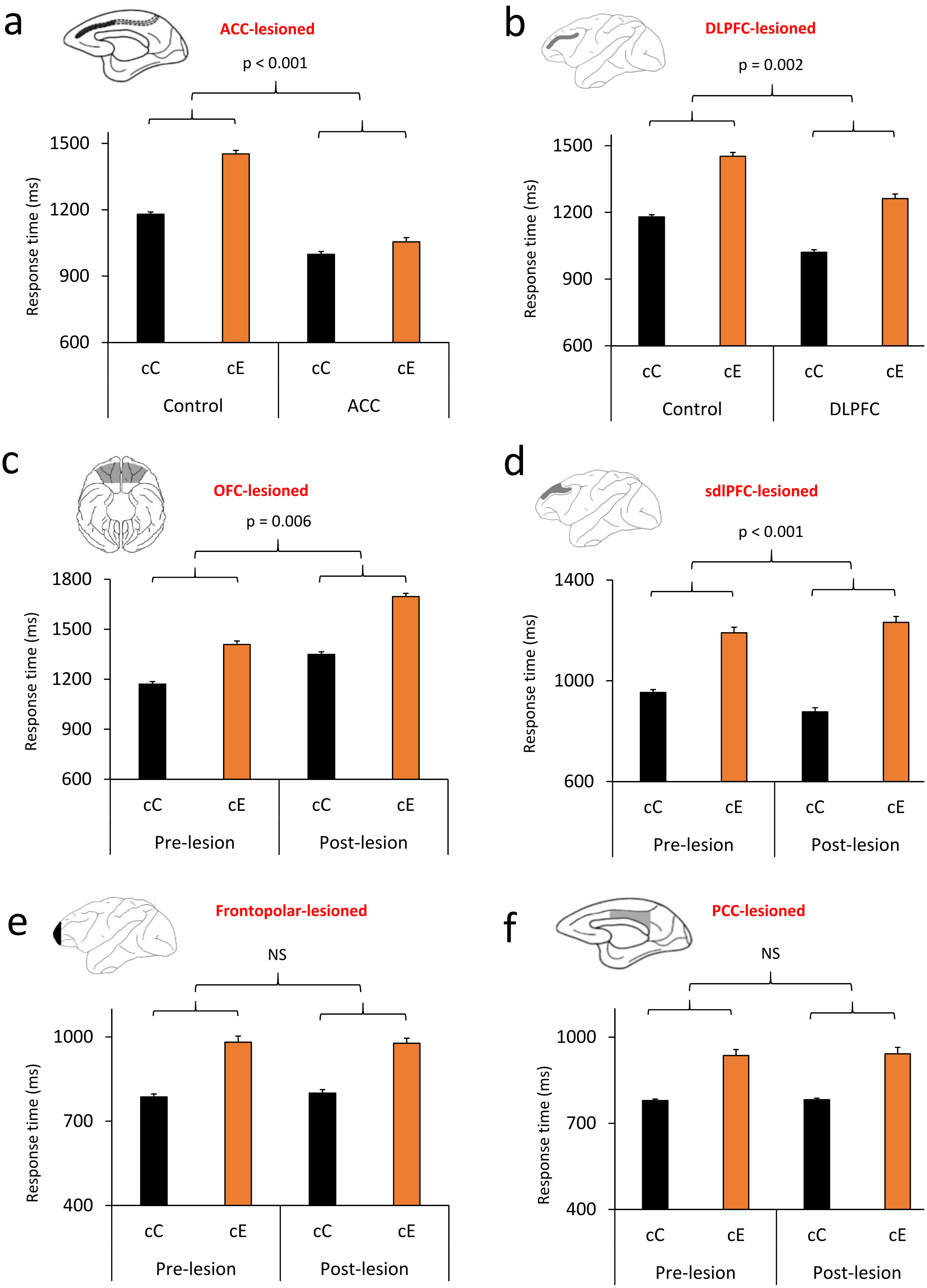
a
ACC-lesioned
p < 0.001
Response time (ms)
1500
1200
900
600
cC
cE
cC
cE
Control
ACC
b
DLPFC-lesioned
p = 0.002
Response time (ms)
1500
1200
900
600
cC
cE
cC
cE
Control
DLPFC
c
OFC-lesioned
p = 0.006
Response time (ms)
1800
1500
1200
900
600
cC
cE
cC
cE
Pre-lesion
Post-lesion
d
sdlPFC-lesioned
p < 0.001
Response time (ms)
1400
1000
600
cC
cE
cC
cE
Pre-lesion
Post-lesion
e
Frontopolar-lesioned
NS
Response time (ms)
1000
700
400
cC
cE
cC
cE
Pre-lesion
Post-lesion
f
PCC-lesioned
NS
Response time (ms)
1000
700
400
cC
cE
cC
cE
Pre-lesion
Post-lesion

Figure S5. Consequence of selective brain lesions on response time in error trials.

The mean response time (RT) is shown in correct (cC) and error (cE) trials, in the same format as those in Figs. 4. (**a**) Anterior cingulate cortex (ACC). (**b**) Dorsolateral prefrontal cortex (DLPFC). (**c**) Orbitofrontal cortex (OFC). (**d**) Superior dorsal-lateral prefrontal cortex (sdlPFC). (**e)** Frontopolar cortex. (**f**) Posterior cingulate cortex (PCC).

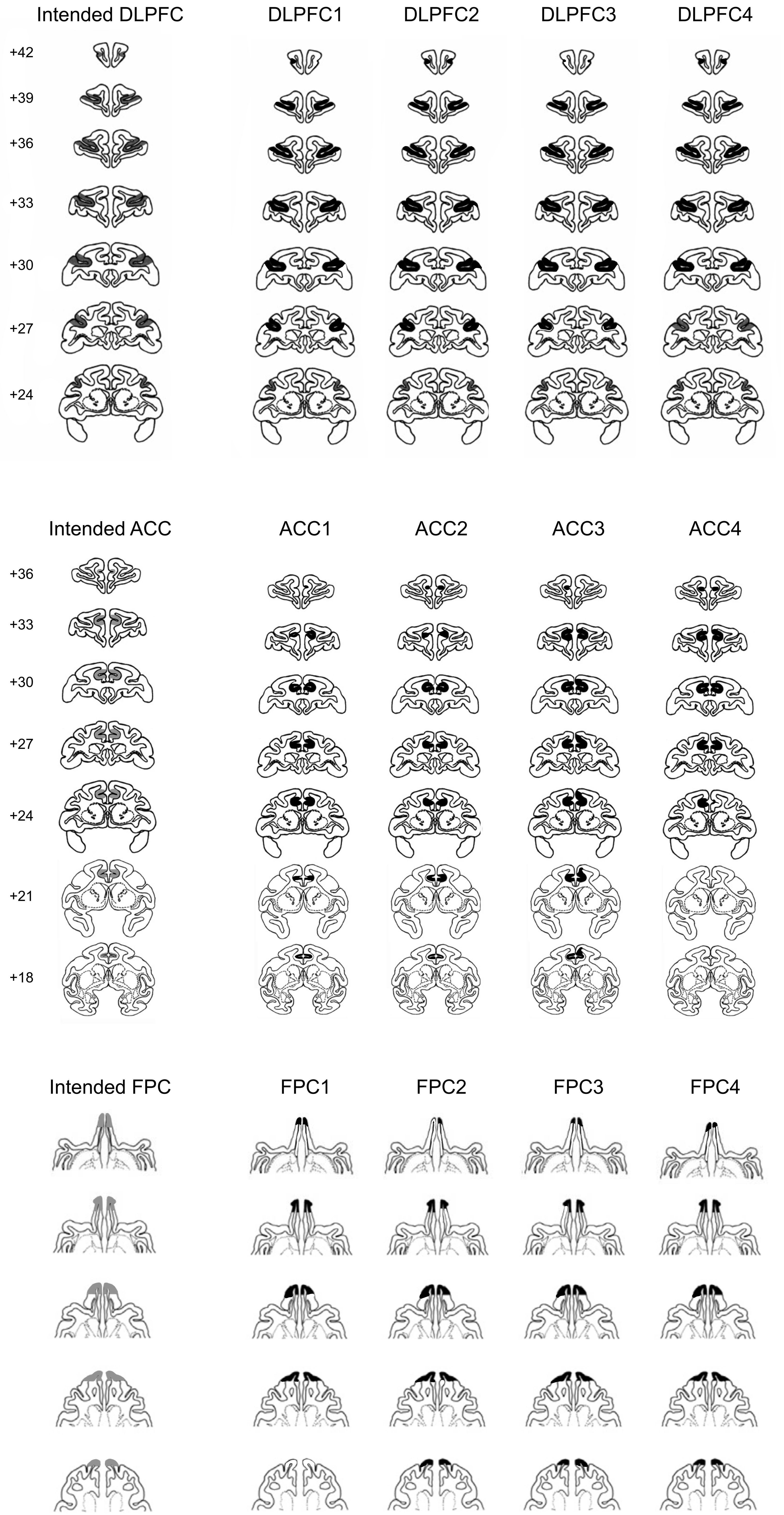
Intended DLPFC
DLPFC1
DLPFC2
DLPFC3
DLPFC4
+42
+39
+36
+33
+30
+27
+24
Intended ACC
ACC1
ACC2
ACC3
ACC4
+36
+33
+30
+27
+24
+21
+18
Intended FPC
FPC1
FPC2
FPC3
FPC4

Figure S6. The extent of lesions in DLPFC, ACC and frontopolar lesion groups.

The schematic shows the intended and actual lesion for the three lesion groups on a standard ventral, lateral, or medial drawing of the macaque brain. For the dorsolateral prefrontal cortex (DLPFC) and anterior cingulate sulcal cortex (ACC) groups the lesions are depicted on a series of drawing of coronal sections (numerals: distance in mm from the interaural plane). For the frontopolar cortex group (FPC), the lesions are depicted on horizontal sections, ventral (top row) to dorsal (bottom row) through the lesion extent. In all cases the intended lesion is shown on the left and the actual lesions for the monkeys in each group depicted in the four right-most columns. In all groups the lesion extent was assessed by microscopic inspection of post-mortem histological sections. Photomicrographs of some stained histological sections have been published previously for the DLPFC and ACC groups as these are the same animals as in that study[1] (Table 1), and similarly, photomicrographs of some stained histological sections have been published previously for the FPC group as these are the same animals as in that study[2] (Table 1). In all cases the actual lesion extents were largely as intended in extent. The lesions were for the most part generally symmetrical with only minor asymmetries inconsistent across subjects; unintended damage to underlying white matter was minor and was not bilaterally symmetrical.

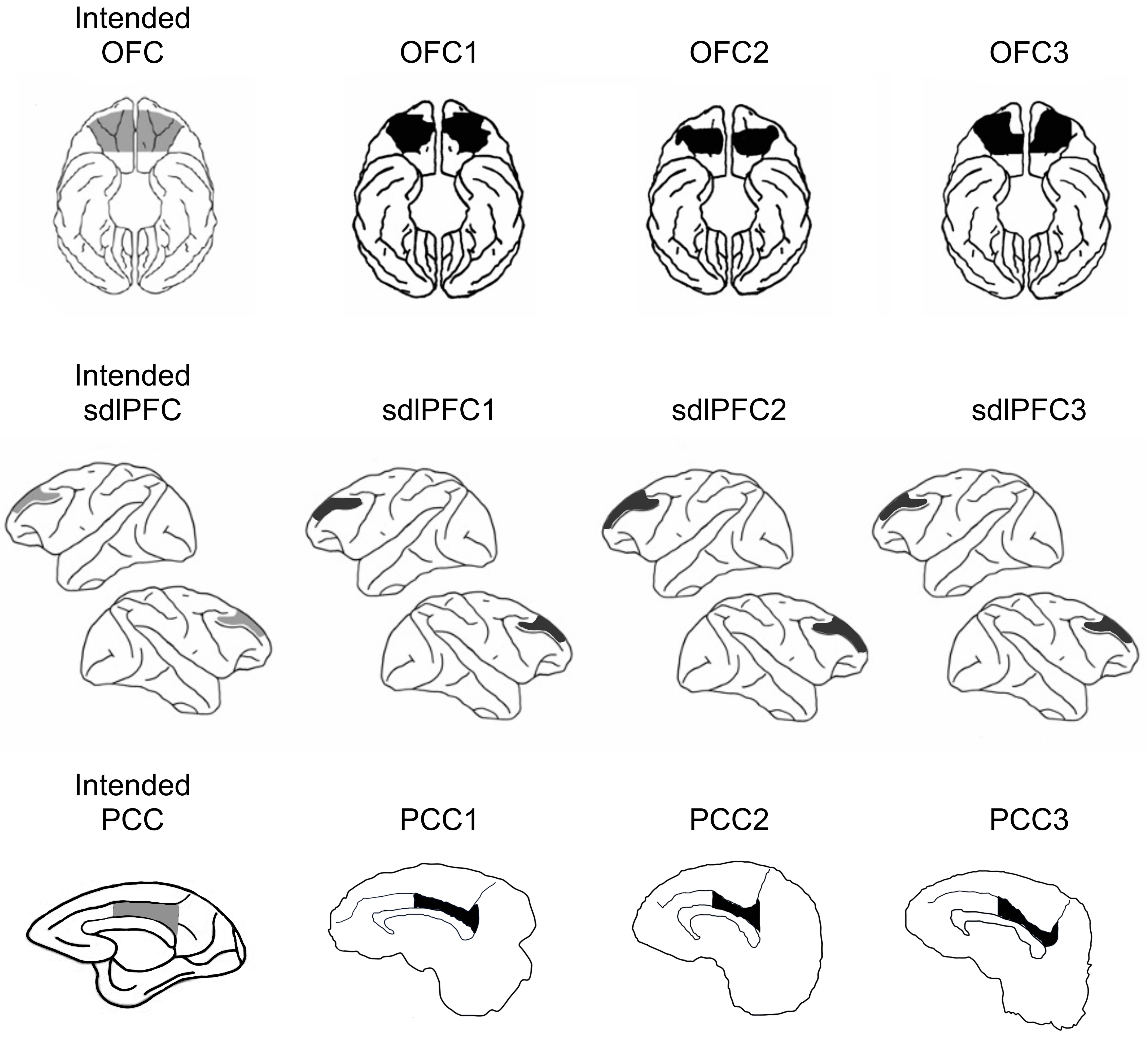
Intended OFC
OFC1
OFC2
OFC3
Intended sdlPFC
sdlPFC1
sdlPFC2
sdlPFC3
Intended PCC
PCC1
PCC2
PCC3

Figure S7. The extent of lesions in OFC, sdlPFC and PCC lesion groups.

The schematics show the intended and actual lesion extent for the three lesion groups whose cortical lesion were not primarily within a sulcus such that the extent of the lesion may be depicted on a standard ventral (orbitofrontal cortex lesion, OFC), lateral (superior dorsolateral prefrontal cortex, sdlPFC), or medial (posterior cingulate cortex, PCC) drawing of the macaque brain. For the OFC, sdlPFC, and PCC groups the intended lesion is shown on the left and the actual lesions for the three monkeys in each group depicted in the three right-most columns. In OFC and sdlPFC groups the lesion extents were assessed by microscopic inspection of post-mortem histological sections. However, in the posterior cingulate cortex group, coronal magnetic resonance images were inspected. Photomicrographs of some stained sections have been published previously for the OFC and sdlPFC groups as these are the same animals as in that study[1] (Table 1), and photomicrographs of post-lesion MRI sections have been published previously for the PCC group as these are the same animals as in that study[2] (Table 1). In all cases, the actual lesion extents were largely as intended in extent. The lesions were for the most part generally symmetrical with only minor asymmetries inconsistent across subjects; unintended damage to underlying white matter was minor and was not bilaterally symmetrical.
