# Supplementary material for: Mapping causal links between prefrontal cortical regions and intra-individual behavioral variability
Source: Nat Commun. 2024 Jan 2;15:140. doi: 10.1038/s41467-023-44341-5 (PMC10762061; doi:10.1038/s41467-023-44341-5)
Supplement: Supplementary file 3 — Reporting Summary [file 41467_2023_44341_MOESM3_ESM.pdf]

nature portfolio

Corresponding author(s): Farshad Alizadeh Mansouri (NCOMMS-23-29832)

Last updated by author(s): Oct 18, 2023

Reporting Summary

Nature Portfolio wishes to improve the reproducibility of the work that we publish. This form provides structure for consistency and transparency in reporting. For further information on Nature Portfolio policies, see our Editorial Policies and the Editorial Policy Checklist.

Statistics

For all statistical analyses, confirm that the following items are present in the figure legend, table legend, main text, or Methods section.

n/a	Confirmed	
☐	☒	The exact sample size (*n*) for each experimental group/condition, given as a discrete number and unit of measurement
☐	☒	A statement on whether measurements were taken from distinct samples or whether the same sample was measured repeatedly
☐	☒	The statistical test(s) used AND whether they are one- or two-sided<br>*Only common tests should be described solely by name; describe more complex techniques in the Methods section.*
☐	☒	A description of all covariates tested
☐	☒	A description of any assumptions or corrections, such as tests of normality and adjustment for multiple comparisons
☐	☒	A full description of the statistical parameters including central tendency (e.g. means) or other basic estimates (e.g. regression coefficient) AND variation (e.g. standard deviation) or associated estimates of uncertainty (e.g. confidence intervals)
☐	☒	For null hypothesis testing, the test statistic (e.g. *F*, *t*, *r*) with confidence intervals, effect sizes, degrees of freedom and *P* value noted<br>*Give P values as exact values whenever suitable.*
☐	☒	For Bayesian analysis, information on the choice of priors and Markov chain Monte Carlo settings
☐	☒	For hierarchical and complex designs, identification of the appropriate level for tests and full reporting of outcomes
☒	☐	Estimates of effect sizes (e.g. Cohen's *d*, Pearson's *r*), indicating how they were calculated

Our web collection on statistics for biologists contains articles on many of the points above.

Software and code

Policy information about availability of computer code

Data collection	CORTEX software (NIMH) was used for controlling the setup and collecting the behavioral data.
Data analysis	Custom-made Matlab programs (Mathworks) were used for extracting behavioral data.<br>SPSS (IBM) statistical package (version 25) was used for statistical analyses.

For manuscripts utilizing custom algorithms or software that are central to the research but not yet described in published literature, software must be made available to editors and reviewers. We strongly encourage code deposition in a community repository (e.g. GitHub). See the Nature Portfolio guidelines for submitting code & software for further information.

Data

Policy information about availability of data

All manuscripts must include a data availability statement. This statement should provide the following information, where applicable:

- Accession codes, unique identifiers, or web links for publicly available datasets
- A description of any restrictions on data availability
- For clinical datasets or third party data, please ensure that the statement adheres to our policy

The full data set for all procedures will be available upon written request to the corresponding author. A source data file, which includes data for all Figures and comparisons in this manuscript has been uploaded.

Research involving human participants, their data, or biological material

Policy information about studies with human participants or human data. See also policy information about sex, gender (identity/presentation), and sexual orientation and race, ethnicity and racism.

Reporting on sex and gender	N/A
Reporting on race, ethnicity, or other socially relevant groupings	N/A
Population characteristics	N/A
Recruitment	N/A
Ethics oversight	N/A

Note that full information on the approval of the study protocol must also be provided in the manuscript.

Field-specific reporting

Please select the one below that is the best fit for your research. If you are not sure, read the appropriate sections before making your selection.

[x] Life sciences [ ] Behavioural & social sciences [ ] Ecological, evolutionary & environmental sciences

For a reference copy of the document with all sections, see nature.com/documents/nr-reporting-summary-flat.pdf

Life sciences study design

All studies must disclose on these points even when the disclosure is negative.

Sample size	For lesion-behavioral study, sample size was determined based on the effect size previously observed with lesions in the prefrontal cortex. In studies with non-human primates, we aim at using the minimum number of animals that would provide reliable and adequate data.
Data exclusions	No data was excluded.
Replication	Pre-lesion and post-lesion data were collected in 15 sessions for each animal in a highly controlled experimental condition to make sure the consistency of data collection.
Randomization	In the first cohort of macaque monkeys, 14 monkeys were trained to perform a computerized version of the WCST (Figure 1A). Then, based on individuals' pre-lesion performance (mean number of rule-shifts in each testing session), the monkeys were assigned to three separate groups of matched abilities. The range and mean of the numbers of pre-lesion shifts between rules were comparable between groups.<br>In the second cohort of monkeys, we trained 7 macaque monkeys to learn the WCST and then based on individuals' pre-lesion performance (mean number of rule-shifts in each testing session), the monkeys were assigned to two separate groups of matched abilities.
Blinding	Investigators were not blind to the presence of lesions because the animal's head was shaved for the purpose of related surgery and therefore at the time of post-lesion testing, blinding was not possible. However, all pre-lesion and post-lesion data collection was done by a computerized setup (in the context of computerized tasks) and followed standard protocol-practice in a highly controlled experimental condition. Therefore, investigators' knowledge about the presence of lesion could not be a confounding factor.

Reporting for specific materials, systems and methods

We require information from authors about some types of materials, experimental systems and methods used in many studies. Here, indicate whether each material, system or method listed is relevant to your study. If you are not sure if a list item applies to your research, read the appropriate section before selecting a response.

Materials & experimental systems

n/a	Involved in the study	
x		Antibodies
x		Eukaryotic cell lines
x		Palaeontology and archaeology
	x	Animals and other organisms
x		Clinical data
x		Dual use research of concern
x		Plants

Methods

n/a	Involved in the study	
x		ChIP-seq
x		Flow cytometry
	x	MRI-based neuroimaging

Animals and other research organisms

Policy information about studies involving animals; ARRIVE guidelines recommended for reporting animal research, and Sex and Gender in Research

Laboratory animals	21 macaque monkeys (7 macaca fuscata and 14 macaca mulatta) were used in this study. We have included Table 1, which includs detailed demographic information for each animal (including age and sex).
Wild animals	No wild animal was used in this study. All animals were transported from experimental breeding facilities.
Reporting on sex	20 males and only 1 female animals were used in this study. The study did not consider sex in analyzing or reporting results.
Field-collected samples	No sample was field-collected.
Ethics oversight	All experimental procedures in Japan conformed to the ethics guidelines specified by RIKEN Centre for Brain Science. All experimental procedures at Oxford University followed the guidelines of the UK Animals (Scientific Procedures) Act of 1986, licensed through the UK Home Office, and approved by Oxford University Committee on Animal Care and Ethical Review.

Note that full information on the approval of the study protocol must also be provided in the manuscript.

Magnetic resonance imaging

Experimental design

Design type	Post-lesion structural (T2 and T1) MRI.
Design specifications	Only structural MRI was conducted.
Behavioral performance measures	Only structural MRI was conducted.

Acquisition

Imaging type(s)	Structural MRI was done for delineating the lesion extent in monkeys with posterior cingulate lesion.
Field strength	4
Sequence & imaging parameters	MP-RAGE (T1 weighted), 0.5mm isotropic voxels, transmit/receive quadrature bird-cage coil
Area of acquisition	The entire brain was scanned for obtaining 3D structural MRI.

Diffusion MRI ☐ Used ☒ Not used

Preprocessing

Preprocessing software	Only structural MRI was conducted. In-house software was used.
Normalization	Four data sets were acquired during the imaging session and averaged post hoc, using in-house software.
Normalization template	Only structural MRI was conducted.
Noise and artifact removal	Only structural MRI was conducted. Four data sets were acquired during the imaging session and averaged post hoc, using in-house software
Volume censoring	Only structural MRI was conducted.

Statistical modeling & inference

Model type and settings	Only structural MRI was conducted.
Effect(s) tested	*Define precise effect in terms of the task or stimulus conditions instead of psychological concepts and indicate whether ANOVA or factorial designs were used.*

Specify type of analysis: ☒ Whole brain ☐ ROI-based ☐ Both

Statistic type for inference (See Eklund et al. 2016)	Only structural MRI was conducted to confirm the lesion extent in animals with posterior cingulate lesion.
Correction	Only structural MRI was conducted to confirm the lesion extent in animals with posterior cingulate lesion.

Models & analysis

n/a	Involved in the study
☒	☐ Functional and/or effective connectivity
☒	☐ Graph analysis
☒	☐ Multivariate modeling or predictive analysis
